# Biodegradable PLGA-*b*-PEG Nanoparticles Induce T Helper 2 (Th2) Immune Responses and Sustained Antibody Titers via TLR9 Stimulation

**DOI:** 10.3390/vaccines8020261

**Published:** 2020-05-29

**Authors:** Kirsty L. Wilson, Gregory P. Howard, Heather Coatsworth, Rhoel R. Dinglasan, Hai-Quan Mao, Magdalena Plebanski

**Affiliations:** 1 School of Health and Biomedical Sciences, Royal Melbourne Institute of Technology (RMIT) University, Melbourne, Victoria 3084, Australia; kirsty.wilson2@rmit.edu.au; 2 Department of Immunology and Pathology, Monash University, Melbourne, Victoria 3181, Australia; 3 Department of Biomedical Engineering, Johns Hopkins School of Medicine, Baltimore, MD 21205, USA; ghowar16@jhu.edu; 4 Institute for NanoBioTechnology, Johns Hopkins University, Baltimore, MD 21218, USA; 5 Emerging Pathogens Institute, Department of Infectious Diseases & Immunology, College of Veterinary Medicine, University of Florida, Gainesville, FL 32611, USA; h.coatsworth@ufl.edu; 6 Department of Materials Science and Engineering, Whiting School of Engineering, Johns Hopkins University, Baltimore, MD 21218, USA

**Keywords:** nanoparticle, adjuvant, vaccine, antibody, immune response

## Abstract

Sustained immune responses, particularly antibody responses, are key for protection against many endemic infectious diseases. Antibody responses are often accompanied by T helper (Th) cell immunity. Herein we study small biodegradable poly (ethylene glycol)-*b*-poly (lactic-co-glycolic acid) nanoparticles (PEG-*b*-PLGA NPs, 25–50 nm) as antigen- or adjuvant-carriers. The antigen carrier function of PEG-*b*-PLGA NPs was compared against an experimental benchmark polystyrene nanoparticles (PS NPs, 40–50 nm), both conjugated with the model antigen ovalbumin (OVA-PS NPs, and OVA-PEG-*b*-PLGA NPs). The OVA-PEG-*b*-PLGA NPs induced sustained antibody responses to Day 120 after two immunizations. The OVA-PEG-*b*-PLGA NPs as a self-adjuvanting vaccine further induced IL-4 producing T-helper cells (Th2), but not IFN-γ producing T-cells (Th1). The PEG-*b*-PLGA NPs as a carrier for CpG adjuvant (CpG-PEG-*b*-PLGA NPs) were also tested as mix-in vaccine adjuvants comparatively for protein antigens, or for protein-conjugated to PS NPs or to PEG-*b*-PLGA NPs. While the addition of this adjuvant NP did not further increase T-cell responses, it improved the consistency of antibody responses across all immunization groups. Together these data support further development of PEG-*b*-PLGA NPs as a vaccine carrier, particularly where it is desired to induce Th2 immunity and achieve sustained antibody titers in the absence of affecting Th1 immunity.

## 1. Introduction

Vaccines to pathogens that require a sustained immune response to be protective in endemic settings often need potent adjuvants or multiple immunizations [1]. The type of immune response desired to be induced by a vaccine largely depends on the pathogen being targeted, and the potential for damaging non-specific reactions in different human populations. Antibodies are critical in targeting extracellular pathogens, whereas cellular immunity, particularly cytotoxic CD8+ T-cells, are required to eliminate intracellular stages of different pathogens. CD4+ T-cell immunity can directly eliminate intracellular pathogens via interferon gamma (IFN-γ) secretion, which promotes intracellular nitric oxide production capable of killing infected cells containing intracellular viruses and parasites [2]. Vaccines for diseases like malaria, Ebola, and tuberculosis require the induction of antibody-mediated and T-cell immune responses for protection. IFN-γ producing cells, known as T helper 1 (Th1) cells, and T helper 2 (Th2) cells produce interleukin 4 (IL-4), which instructs B cells to produce antibodies, as IL-4 is a known co-mitogen of B cells, and a key factor for the survival of multiple immune cell types in B cell germinal centers [3,4].

The nature of the CD4+ T-cell immune response towards a specific antigen needs to be carefully calibrated, given that some individuals develop autoimmune Th1 mediated pathologies, whilst others may be prone to suffer exacerbation from a Th2 response, such as individuals who suffer from allergic inflammation [5]. Nanoparticles and microparticles have emerged as a powerful vaccine delivery system by increasing uptake of the target antigen at specific sites or by activating specific antigen presenting cell (APC) populations, such as dendritic cells [6]. Particle properties such as size, surface characteristics, and composition can be manipulated to promote different types of antigen presentation and cellular uptake, as well as subsequent immune responses [7]. For example, polystyrene nanoparticles (PS NPs) in the 40–50 nm viral size range elicit high levels of CD4+ and CD8+ T-cells, and long-lasting antibody responses [8,9].

Whilst PS NPs serve as an excellent benchmark for nanoparticle vaccine carriers, their translational utility is limited by its non-degradable nature. Selecting biocompatible and biodegradable nanoparticles (NPs) with similar size and surface characteristics could provide new opportunities for enabling delivery of viral or bacterial mimicking NP-antigen conjugates to promote self-boosting immune responses. We have utilized a flash nanoprecipitation (FNP) process which allows for the generation of 20–50 nm sized nanoparticles using diblock amphiphilic polymers. We have previously used poly(ethylene glycol)-*block*-poly(lactic-*co*-glycolic acid) (PEG-*b*-PLGA) polymers to generate biodegradable NPs with average sizes of 20-, 50-, and 100-nm with minimal size overlap using the FNP method [10]. We demonstrated that 20-nm NPs most effectively transport to, and retention within, the local draining lymph nodes after subcutaneous (*s.c.*) administration [10]. Immunofluorescent staining revealed that the 20- and 50-nm PEG-*b*-PLGA NPs could penetrate into the paracortex and gain access to the abundant population of immature CD11c+ dendritic cells, making these NPs highly attractive for delivering lymph node targeting vaccines [10].

Emerging NP-based vaccines often include additional adjuvants into the formulation, which stimulate innate danger-signal receptors, such as Toll-like receptor (TLR) agonists [11]. The most widely used TLR agonists are ligands for extracellular TLR4, i.e., lipopolysaccharide (LPS) and its non-toxic derivative monosphosphoryl lipid A (MPL-A) [12,13], and those that target intracellular TLRs such as CpG, the ligand for TLR9 [14,15,16]. Ligands targeting intracellular TLRs are increasingly popular due to their ability to induce antibody responses, as well as T-cell responses which promote high affinity antibody production, modulate antibody isotypes, and promote long lasting immunity [17]. The way that the TLR9 signal is delivered, the intracellular endosomal compartments it targets, and the strength and multi-valency of the signal all collectively control the ability of APCs to stimulate other cell types [18,19]. Therefore, NPs with surface-conjugated or releasable CpG may serve as a carrier to deliver this signal at the optimized density and strength to a desired intracellular compartment.

The biophysical presentation of CpG oligodeoxynucleotides (ODNs) greatly influences immunological results. CpG oligomerization plays a key role in controlled self-assembly of D-type CpG ODNs into multimers (Class A), which stimulates plasmacytoid dendritic cells and facilitates IFN-α induction, whereas K-type CpG ODNs (Class B) mainly stimulate B cells by activating interleukin 6 (IL-6) production whilst being weak IFN-α inducers [19,20,21]. This oligomerization can be controlled using NPs of controlled surface CpG density. Using PLGA NPs and microparticles of various CpG 1826 density, Leleux et al. demonstrated that NPs with a high surface density of CpG ODNs (1.49 mg CpG/m^2^) generated a Th1 response while the same NPs with a low surface density of CpG ODNs (0.4 mg CpG/m^2^) gave a Th2-biased response [22]. Fine differences in viral-sized NPs with the same CpG surface density have likewise been shown to be a key factor for immunostimulation of TLR9 by CpG, where 50-nm Au spherical NPs had greater uptake and immunostimulatory potential compared to 13-nm Au spherical NPs [23]. With these insights in mind, we designed a low CpG surface density and a 40–50 nm NP size range for boosting the Th2 response. For this study, we utilized CpG ODN 1018, a Class B CpG developed by Dynavax Technologies that was FDA approved for human use in a Hepatitis B vaccine (HEPLISAV-B™) in 2018 [24]. This CpG ODN 1018 has been found to boost the onset of protective antibody titer, induce superior antibody response, and induce a higher responder rate in healthy, elderly, and immunocompromised patients [25,26].

Herein we investigate whether PEG-*b*-PLGA NPs can induce high level and long-lasting antibody responses, and whether theses NPs can concomitantly induce antigen specific CD8+ or CD4+ T-cell responses. To address this in a fundamental study, we used the well-known model antigen ovalbumin (OVA), which contains well-characterized B-cell, CD4+ and CD8+ T-cell epitopes. Moreover, we benchmarked the results against the experimental PS NP vaccine formulation. We also tested CpG surface conjugation on size-controlled PEG-*b*-PLGA NPs for their potential as a co-administered adjuvant to enhance antibody, CD4+ and CD8+ T-cell induction, when mixed with either protein antigen, OVA-conjugated PS NPs, or biodegradable OVA-conjugated PEG-*b*-PLGA NPs.

## 2. Materials and Methods

### 2.1. Preparation and Characterization of PEG-b-PLGA NPs

**Figure 1:**
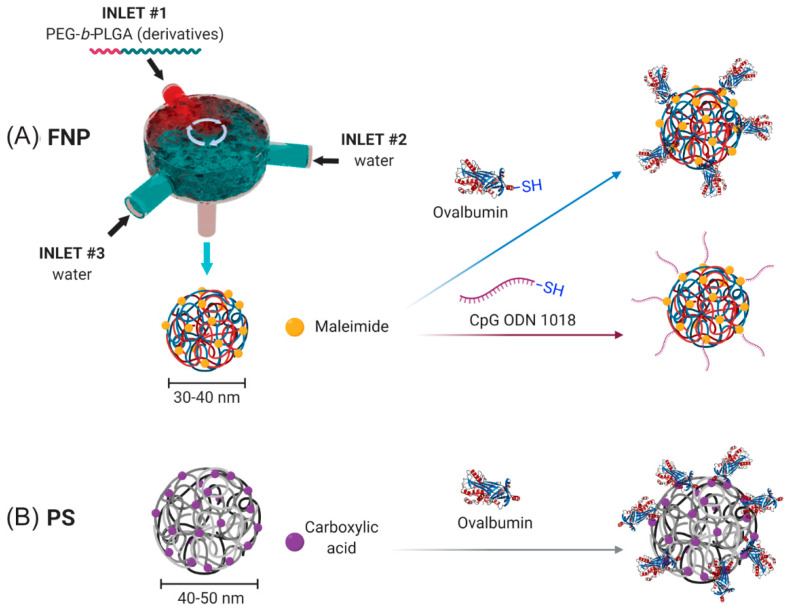
Nanoparticle conjugation schema. (**A**) Conjugation scheme for flash nanoprecipitation (FNP) produced PEG-*b*-PLGA NPs using maleimide-thiol chemistry for surface conjugation of thiolated ovalbumin (OVA) or CpG oligodeoxynucleotide (ODN) 1018. (**B**) Conjugation scheme for PS NPs using EDC NHS ester chemistry.

NPs were generated using a FNP process (Figure 1A) as previously described [10]. Briefly, NPs were generated using a three-inlet, confined impinging jet (CIJ) FNP device. The first two inlet inputs contained distilled, deionized (DDI) water. The third inlet contained a solution of 5 mg/mL PEG_3K_-*b*-PLGA_20K_ containing 90 mol% unlabeled PEG_3K_-*b*-PLGA_20K_ (Polyscitech) and 10 mol% maleimide-PEG_3K_-*b*-PLGA_20K_ (Polyscitech) dissolved in acetonitrile. NPs of the defined size were generated by modulating volumetric flow rates of the three inlets using a NE-4000 Programmable 2 Channel Syringe Pump (New Era Pump Systems). The NPs generated through the device were collected in a water bath such that ≤10% *v*/*v* was acetonitrile. The collected NPs were dialyzed in a 3.5-kDa dialysis membrane (Repligen) at 4 °C against 4 L of deionized water with dialysate changes every six hours.

### 2.2. Surface Conjugation of OVA to PS and PEG-b-PLGA NPs

Conjugation of chicken egg ovalbumin (OVA) to Mal-PEG-*b*-PLGA NPs was performed as follows. Briefly, EndoFit OVA (Sigma Aldrich) was thiolated (OVA-SH) using the Traut’s reagent (Sigma Aldrich) according to the manufacturer’s protocol at a 14.2× molar excess. The thiolated OVA was dialyzed overnight in 0.1× PBS, pH 7.0 to remove excess Traut’s reagent, with two dialysis changes every 6 h. The thiolated OVA was then treated with tris(2-carboxyethyl) phosphine hydrochloride (TCEP) according to the manufacturer’s protocol (Sigma Aldrich) for 1 h to remove disulphide bridges between OVA molecules. The 20–30 nm PEG-*b*-PLGA NPs with 10 mol% maleimide-PEG-*b*-PLGA in 0.1× PBS pH 6.2–6.5 buffer were mixed with EndoFit OVA-SH overnight at a maleimide:SH ratio of 3:1 for 16 h at 4 °C. NPs were then collected, washed ten times to remove unreacted OVA, and concentrated at 400× *g* at 5 min intervals using an Amicon^®^ 100-kDa MWCO centrifugal filter (EMD Millipore) and a Sorvall RT1 Centrifuge (Thermofisher Scientific) until the desired concentration was reached. OVA concentration of the NP solutions was quantified using a Pierce microBCA protein assay and diluted to achieve the desired stock concentrations.

Conjugation of OVA to PS NPs was performed as described previously [27,28]. Briefly, 40–50 nm carboxylated PS nanoparticles were pre-activated using a 2-N-morpholino-ethanesulfonicacid buffered (MES; 50 mM, pH 6.2) solution of 1-ethyl-3-(3-dimethylaminopropryl) carbodiimide hydrochloride (EDC; 4 mg/mL) (Sigma Aldrich) (with sulfo-NHS for some conjugations) for 1 h on a rotating wheel at room temperature. OVA was added (target final concentration of 1 mg/mL) and further incubated for 2 h at room temperature. The conjugation reaction was stopped by the addition of glycine (7 mg/mL) for a further 30 min. The conjugation mixture was dialyzed using a 100-kDa dialysis membrane overnight against phosphate buffered saline (PBS) at 4 °C. The conjugated PS NPs were stored at 4 °C and sonicated for 15 min before use to create a homogenous solution for immunization.

### 2.3. Surface Conjugation of CpG to PEG-b-PLGA NPs

Conjugation of CpG to PEG-*b*-PLGA NPs was performed as follows. CpG ODN 1018 5′-thiol S-S C6- TGACTGTGAACGTTCGAGATGA-3′ with a phosphorothioate backbone was synthesized by TriLink Biotechnologies. To remove the 5′ C6 thiohexyl modification from CpG 1018 and to present a reactive thiol terminus for NP conjugation, 400 µL of 0.1 M TCEP in sterile RNase free water was added to lyophilized thiolated CpG 1018 for 1 h to reduce the thiol groups under intermittent vortexing. After 1 h, 50 µL of 3 M sodium acetate was added and vortexed, and 1.5 mL of absolute ethanol was added. The solution was then vortexed and stored at −20 °C for 20 min. The sample was then centrifuged at 12,000 rpm for 10 min. The ethanol was decanted and allowed to air dry in a sterile cell culture cabinet. The CpG 1018 pellet was dissolved in 200 µL of sterile RNase free water and the sample concentration was obtained by measuring UV-absorbance at 260 nm. To reduce the terminal thiol groups on UF6 peptide, it was treated with 10× molar excess of TCEP in DI water for 1 h at room temperature under intermittent vortexing. The excess TCEP was removed by dialysis of UF6 peptide using a 3.5-kDa MWCO dialysis membrane and degassed with 4 L 0.1× PBS with 2 mM EDTA at pH 6.2–6.5 with changes every 3 h.

Conjugation of CpG to PEG-*b*-PLGA NPs followed a similar protocol as above. The NP solution at a concentration of 300 µg/mL was collected and reacted with CpG-SH at a maleimide:SH ratio of 2:1 for 16 h at 4 °C. NPs were then collected, washed 10× to remove unreacted CpG, and concentrated at 400× *g* at 5 min intervals using a 100-kDa MWCO centrifugal filter and a Sorvall RT1 centrifuge (Thermo Fisher) until the desired concentration was reached. CpG concentration of the NP solutions was determined using a NanoDrop 1000 spectrophotometer (Thermo Fisher) and diluted to achieve the desired NP concentration.

### 2.4. Characterization, Lyophilization, and Reconstitution of OVA-PEG-b-PLGA NPs and CpG-PEG-b-PLGA NPs

Post-fabrication and concentration, each NP formulation was diluted to 100 μg/mL NP in 10 mM HEPES buffer containing 10 mM NaCl (pH 7.2) and its NP hydrodynamic diameter (number-average) were measured by Dynamic Light Scattering (DLS) using a Malvern Zetasizer Nano ZS. The morphology and dry size of each NP was determined using transmission electron microscopy (TEM) using a Technai FEI-12 TEM electron microscope. Samples were further diluted and 20 μL aliquots were absorbed onto an ionized nickel grid covered with carbon films for 30 min at room temperature. The NP suspension was removed by blotting using Whatman filter paper. The samples were then stained with 2% (*w*/*v*) uranyl acetate for 45 s. The grids were blotted to remove excess negative stain and allowed to dry in a chemical hood overnight prior to imaging.

OVA-PEG-*b*-PLGA NPs and CpG-PEG-*b*-PLGA NPs were directly mixed with 20% *w*/*v* sucrose cryoprotectant to yield the desired dose of antigen/adjuvant with a final concentration of 10% *w*/*v* sucrose. The NPs were aliquoted by 300 µL into sterile 2.0 mL screw-top tubes, frozen overnight using a Nalgene^®^ Mr. Frosty in a −80 °C freezer and lyophilized using a FreeZone Triad Benchtop Freeze Dryer (LABCONCO, Kansas City, USA). The lyophilized powder was stored in a −80 °C freezer until use. To reconstitute the NPs, 300 µL of DI water was added and vortexed for 10 s. NPs were then immediately administered by intradermal (*i.d.*) administration.

### 2.5. Animal Care and Use Approval

All animal experiments were approved by the Alfred Medical Research and Education Precinct (AMREP) animal ethics committee and performed in accordance with the animal code of conduct (E/1454/2014/M). Six-to-eight-week old C57BL/6 mice were purchased from Monash Animal Services.

### 2.6. Immunization Protocol

Mice were immunized intradermally at the base of the tail twice, 14 days apart, with one of the following formulations: PBS only (naïve), OVA alone, OVA mixed with CpG-conjugated PEG-*b*-PLGA NPs (OVA + CpG-PEG-*b*-PLGA NPs), OVA-conjugated PS NPs (OVA-PS NPs), OVA-PS NPs with CpG-conjugated PLGA-*b*-PEG NPs (OVA-PS NPs + CpG-PEG-*b*-PLGA NPs), OVA-conjugated to PEG-*b*-PLGA NPs (OVA-PEG-*b*-PLGA NPs), or OVA-PEG-*b*-PLGA NPs plus CpG-PEG-*b*-PLGA NPs (OVA-PEG-*b*-PLGA NPs + CpG-PEG-*b*-PLGA NPs). The OVA concentration immunized for all NP formulations was 50 µg OVA/mouse. The CpG groups received 5 µg CpG/mouse.

### 2.7. ELISA to Measure OVA-Specific Antibody Titer

Blood from immunized animals was collected by a submandibular bleed at Days 0, 14, 28, 49, 91, and at the endpoint Day 120 by a cardiac bleed. Sera were harvested for use in IgG ELISA assays. A 96-well plate (Nunc Maxisorp plates) was coated with 5 μg/mL of OVA in carbonate/bicarbonate coating buffer (pH 9.6) overnight at 4 °C, and washed with 0.05% Tween 20/PBS, and then blocked with 5% skim milk/PBS by incubation for 1 h at 37 °C. The plate was washed again with 0.05% Tween 20/PBS and kept at 4 °C in PBS before use. Sample sera from different time points were serially half-log diluted (from 500× to 1,093,500×) and added to the wells of the pre-coated plate, and then incubated at 4 °C overnight. The plate was washed with 0.05% Tween 20/PBS, and horseradish peroxidase (HRP)-conjugated anti-mouse total IgG antibody (1/2000 dilution, goat anti-mouse IgG (H + L), Invitrogen) was added and incubated for 2 h at 37 °C. The plate was given a final wash and antibody detection was developed using TMB substrate solution before being stopped with 1 N HCl. Absorbance was read at an optical density (OD) of 450 nm on a plate reader (Multiscan GO, Thermo Fisher). Antibody titers were calculated closest to the serum dilution at which the OD was equal to the mean of the naïve sera (averaged across naïve mice for all dilutions tested) plus 3 standard deviations.

### 2.8. ELISpot to Measure OVA-Specific T-Cell Response

Splenocytes from immunized animals were isolated 14 days following the second immunization and assessed by ELISpot for IFN-γ and IL-4 production. Ninety-six-well multiscreen plates (MSIP, Millipore) were coated overnight at 4 °C with 5 μg/mL anti-mouse IFN-γ (AN18, Mabtech) or anti-mouse IL-4 (BVD4-1d11, BD Biosciences). All wells were washed 5 times with PBS and blocked with RPMI 1640 (Gibco, Life Technologies) containing 10% FBS (further supplemented with 100 units/mL penicillin, 100 μg/mL streptomycin, 4 mM L-glutamine, 1 M HEPES, and 0.1 mM 2-mercaptoethanol, to make complete media) for a minimum of 1 h at 37 °C. Splenocytes were added at a final concentration of 0.5 × 10^6^ per well and co-incubated with recall antigens (OVA at a final concentration of 25 µg/mL, CD4-specific OVA epitope (ISQAVHAAHAEINEAGR) at a final concentration of 25 µg/mL and CD8-specific OVA epitope (SIINFEKL) at a final concentration of 2.5 µg/mL) in complete media for ~16 h for IFN-γ plates and ~28 h for IL-4 plates at 37 °C. Media alone and positive control Concanavalin A (1 μg/mL final) wells were also added. Following incubation, plates were washed with PBS and then 1 μg/mL anti-mouse IFN-γ-biotin (R4-6A2, Mabtech) or anti-mouse IL-4-biotin (BVD6- 24G2, Mabtech or BD Biosciences) in 0.5% FBS/PBS was added for 2 h at RT. Plates were washed and 1 μg/mL Streptavidin-ALP or extravidin-ALP in 0.5% FBS/PBS was added for a further 1.5 h incubation at room temperature. Plates were given a final wash with PBS followed by reverse osmosis water and spots were developed using an AP colorimetric kit (Bio-Rad, Hercules, USA) following the manufactures instructions. Once plates were dry, spots were counted using an AID ELISpot Reader system (AutoImmune Diagnostika, GmbH, Straßberg, Germany).

### 2.9. Statistical Analyses

End-point titer data were analyzed by a Kruskal–Wallis test followed by pairwise Mann–Whitney post-hoc tests. Analyses were completed in R (v. 3.6.2) at an α = 0.05 [29]. Linear regression analyses were completed in Prism (v. 8.4.0).

## 3. Results

The FNP platform allowed for generation of Mal-PEG-*b*-PLGA NPs with a number average size of 28.9 ± 1.8 nm and polydispersity index (PDI) of 0.20 ± 0.01 indicating a high level of uniformity as confirmed by TEM imaging (Figure S1), corroborating with our previously published results [10]. Conjugation of OVA onto the NP surface resulted in a 14-nm increase in size (OVA-PEG-*b*-PLGA NP size: 43.6 ± 1.0 nm, PDI: 0.19 ± 0.02), consistent with conjugation of OVA (2.9 nm hydrodynamic diameter) and peak broadening (Figure S1A). Inclusion of CpG on the NP surface resulted in no apparent increase in NP size, due to the small size of CpG (CpG-PEG-*b*-PLGA NP average size: 29.4 ± 8.0 nm, PDI: 0.30 ± 0.03). The small NP size (<50 nm) and high uniformity (PDI < 0.3) for all groups confirmed the suitability of these NPs for lymph node targeting and immunization after *i.d.* administration [10].

The enhancement effect of PS NPs as an antigen carrier has been well established in previous studies [8,9,27,30,31]; therefore they were used as an experimental benchmark to assess the ability of PLGA-*b*-PEG NPs as a vaccine carrier. All testing formulations were given to two cohorts of mice intradermally at the base of the tail twice at Days 0 and 14. Fourteen days following the second immunization, one cohort of mice were sacrificed, and splenocytes were harvested for examining the induced T-cell response by ELISpot. The other cohort was kept for another three months and periodically bled to assess the long-term antibody response to each formulation.

Most prophylactic vaccines need a strong antibody response to target extracellular pathogens and ideally will induce the production of stable long-term antibodies following a minimal immunization regimen. To assess antibody response over time, one cohort of mice were immunized twice as above, and bled at the follow day (D) time points: D0, D14, D28, D49, D91, and D120. The pre-bleed D0 time point showed no response for any group, as anticipated (Figure S2). There were also very little antibody responses at D14, after a single immunization, with all groups requiring a boost immunization to produce higher antibody levels. At D28, two weeks following the second immunization, all groups showed that mice were responding to the vaccines, with IgG antibodies detected for most mice from D28 until the end time point of D120 (Figure S2). From D28, the OVA alone group also showed transient IgG responses above background, which returned to basal levels at day 120 (Figure S2). OVA-PEG-*b*-PLGA NPs immunized mice displayed consistent IgG antibody production in three out of the four mice across all time points following two immunizations, including D120; whereas responses to OVA-PS NPs were less consistent, with two of the four mice immunized with OVA-PS NPs showing a very high antibody response, but two mice responding weakly (D28 through to D120) (Figure S2).

**Figure 2:**
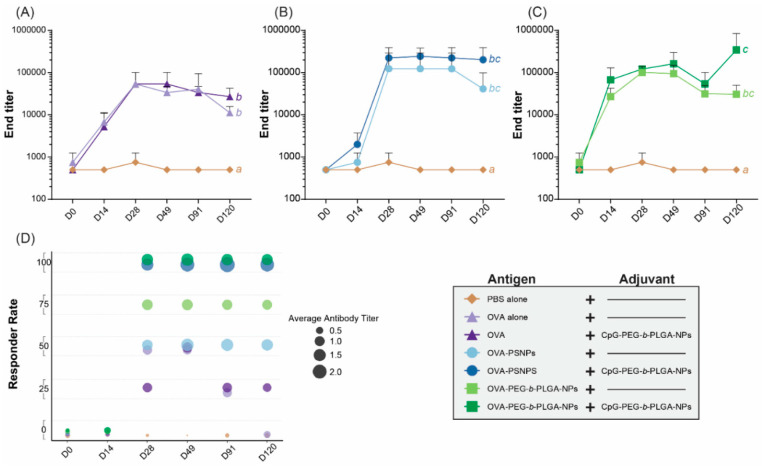
Antibody end titers over time. C57BL/6 mice were immunized twice, two weeks apart, intradermally at the base of the tail with the following formulations; PBS alone (Naïve), OVA alone, OVA + CpG-PEG-*b*-PLGA nanoparticles (NPs), OVA-PS NPs, OVA-PS NPs + CpG-PEG-*b*-PLGA NPs, OVA-PEG-*b*-PLGA NPs, or OVA-PEG-*b*-PLGA NPs + CpG-PEG-*b*-PLGA NPs. Treatments without a common letter were found to be statistically significant (α = 0.05) via a Kruskal–Wallis test with Mann–Whitney pairwise post-hoc comparisons. Comparison of (**A**) OVA alone with and without CpG-PEG-*b*-PLGA NPs, (**B**) OVA-PS NPs with and without CpG-PEG-*b*-PLGA NPs, and (**C**) OVA-PEG-*b*-PLGA NPs with or without CpG-PEG-*b*-PLGA NPs. All animals received 50 µg OVA. For CpG-PEG-*b*-PLGA NP groups, mice received 5 µg CpG. Graphs show average end titers for each group at each day (**D**) timepoint, D0, D14, D28, D49, D91, and D120. End titer was calculated as the closest to average of naïve mice + 3 SD, results shown as average ± SD for each time point (n = 4 mice per group). (**D**) Responder rate (%, n = 4 mice per group) and average antibody titer for each immunogen treatment as defined as OD420 above 1.0 at dilution 1500 (from Figure S2).

The addition of the CpG- PEG-*b*-PLGA NPs to the formulations led to a more sustained and consistent response across all groups, with all mice receiving the adjuvant NPs displaying higher levels of antibody responses above those of naïve animals from D28 to D120 (Figure S2, Figure 2). The end titers across all time points revealed that the addition of CpG-PEG-*b*-PLGA to OVA-PS NPs in the observed response was higher than the OVA-PS NPs from D28 through D120 (Figure 2B). A similar effect was observed for OVA-PEG-*b*-PLGA NPs where addition of CpG-PEG-*b*-PLGA NPs boosts the antibody response at D14, D49 and D120 (Figure 2C). The groups receiving the addition of CpG-PEG-*b*-PLGA NPs had higher titers at the endpoint of the study (D120). The addition of CpG-PEG-*b*-PLGA NPs consistently increased antibody responses (Figure 2B,C) and responder rates in the OVA-PS NP and OVA-PEG-*b*-PLGA NP groups (Figure 2D). All the treatment groups were found to elicit a significantly different response as compared to the naïve, PBS control (*p* < 0.05); however, only the formulation with OVA-PEG-*b*-PLGA NPs + CpG-PEG-b-PLGA NPs was significantly different than the OVA alone and OVA + CpG-PEG-*b*-PLGA NP groups (*p* < 0.05) (Table S1).

**Figure 3:**
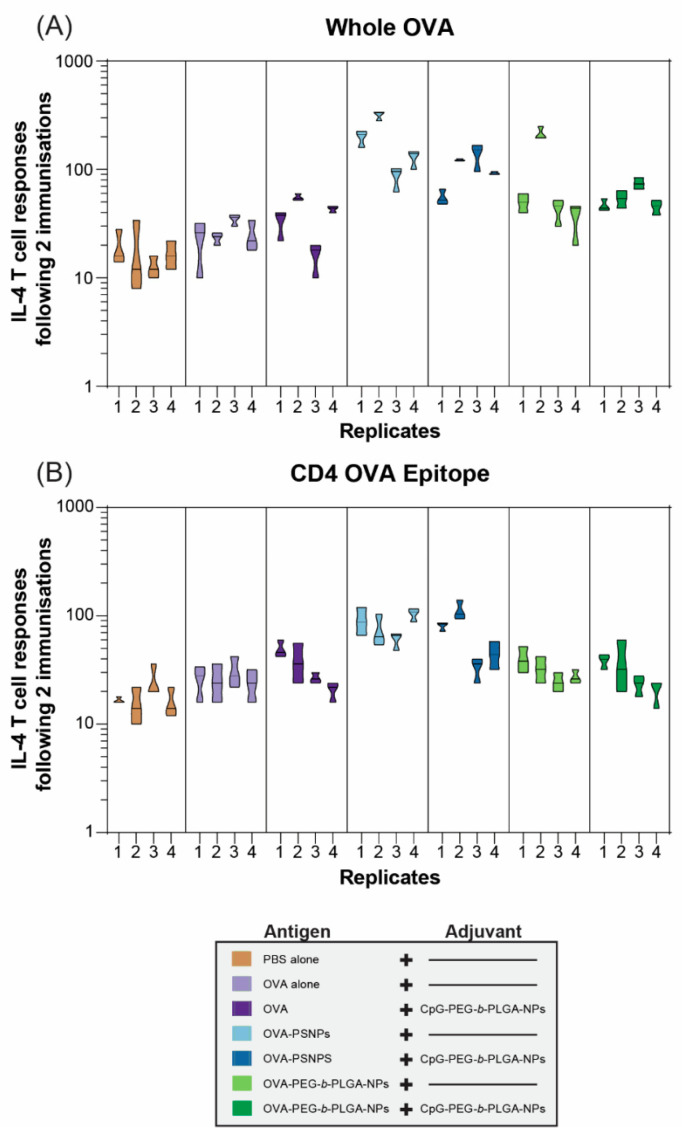
Interleukin 4 (IL-4) T-cell responses to nanovaccine formulations at day 28. C57BL/6 mice were immunized twice, two weeks apart, intradermally at the base of the tail with the following formulations; phosphate buffered saline (PBS) alone (Naïve), OVA alone, OVA + CpG-PEG-*b*-PLGA NPs, OVA-PS NPs, OVA-PS NPs + CpG-PEG-*b*-PLGA NPs, OVA-PEG-*b*-PLGA NPs, or OVA-PEG-*b*-PLGA NPs + CpG-PEG-*b*-PLGA NPs. All animals received 50 µg OVA. CpG-PEG-*b*-PLGA groups received 5 µg CpG. Graph shows IL-4 T-cell responses to (**A**) whole OVA and the (**B**) CD4+ T-helper OVA epitope by ELISpot assay. Individual mice (replicates 1–4 for control and treatment groups) are shown for each recall antigen ± experimental error from triplicate assay wells.

Robust and sustained antibody responses rely on T follicular helper (Tfh) cells and IL-4 to produce high affinity, class switched, IgG antibodies [3,32]. As such, we examined the functional IL-4 T-cell response via the ELISpot assay in one cohort of mice at two weeks after the second immunization (D28). OVA immunization alone failed to promote a Th2 immune response; and this was improved marginally by the addition of CpG-PEG-*b*-PLGA NPs: Only 2 out of 4 immunized animals showed a 2-fold increase in reactivity compared to the negative control PBS-immunized mice (Figure 3A). In contrast, OVA-PS NPs induced over 2-fold increases in IL-4 T-cell responses to whole OVA in all animals, including one animal, which reached a >10-fold increase in reactivity. The addition of CpG-PEG-*b*-PLGA NPs did not further increase this response. Responses induced by OVA-PEG-*b*-PLGA NPs were found in 3 of 4 animals reaching more than 2-fold increases, including a super-responder with an over 10-fold increase. Addition of CpG-PEG-*b*-PLGA NPs did not further increase this response. The overall IL-4 response to the CD4+ T-cell epitope tested as a minimal peptide epitope by ELISpot was lower than the response to whole OVA, with the most consistent responses displayed by the OVA-PS NP group (Figure 3B). Two of the four mice in the group received OVA-PS NPs plus CpG-PEG-*b*-PLGA NPs which induced a moderate level of IL-4 to OVA-specific CD4+ T-cells recognizing the epitope ISQAVHAAHAEINEAGR. There was only a moderate response detected (2-fold increase vs. PBS control mice) in 2 out of 4 of the animals immunized with OVA-PEG-*b*-PLGA NPs alone, or with the addition of CpG-PEG-*b*-PLGA NPs.

**Figure 4:**
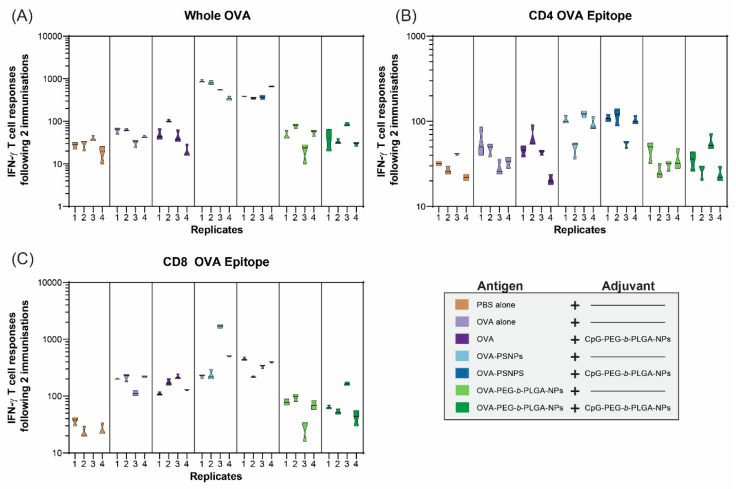
Interferon gamma (IFN-γ) T-cell responses to nanovaccine formulations at Day 28. C57BL/6 mice were immunized twice, two weeks apart, intradermally at the base of the tail with the following formulations; PBS alone (Naïve), OVA alone, OVA + CpG-PEG-*b*-PLGA NPs, OVA-PS NPs, OVA-PS NPs + CpG-PEG-*b*-PLGA NPs, OVA-PEG-*b*-PLGA NPs, or OVA-PEG-*b*-PLGA NPs + CpG-PEG-*b*-PLGA NPs. All animals received 50 µg OVA. CpG-PEG-*b*-PLGA NP groups received 5 µg CpG. Graph shows IFN-γ T-cell responses to (**A**) whole OVA, (**B**) CD8+ T-cell epitope (SIINFEKL), and the (**C**) CD4+ T-helper OVA epitope (ISQAVHAAHAEINEAGR) by ELISpot assay. Individual mice are shown for each recall antigen ± experimental error from triplicate assay wells.

In addition to IL-4 T-cell responses, IFN-γ was assessed to determine whether the OVA-PEG-*b*-PLGA NPs were inducing a Th1 response (Figure 4). IFN-γ is the most common cytokine assessed for vaccine studies as a Type 1 (Th1) response from T-cells, and also to examine the response to CD8+ T-cell epitopes, such as SIINFEKL in OVA. As expected based on previous studies [31,32], the OVA-PS NPs elicited a high level of IFN-γ producing T-cells specific to OVA from all immunized mice, as well as from mice immunized with OVA-PS NPs plus CpG-PEG-*b*-PLGA NPs (Figure 4A). The addition of the CpG-PEG-*b*-PLGA NPs did not change the response to OVA compared to OVA-PS NPs alone. The OVA-PEG-*b*-PLGA NPs did not induce IFN-γ to whole OVA above background levels, nor did the OVA-PEG-*b*-PLGA NPs plus CpG-PEG-*b*-PLGA NPs.

The IFN-γ responses to the CD4+ T-cell epitope (OVA323–339, ISQAVHAAHAEINEAGR) were much lower overall, compared to whole OVA, with minimal responses above background (Figure 4B). The only notable responses were from three out of four mice in the two groups that received OVA-PS NPs and OVA-PS NPs and CpG-PEG-*b*-PLGA NPs, respectively. Both groups induced low levels of IFN-γ responses to the CD4+ T-cell epitope that were of similar magnitude for each group. In contrast, we observed high levels of IFN-γ responses to the CD8+ T-cell epitope (SIINFEKL) by the OVA-PS NPs, including one mouse with an extremely high response (Figure 4C). The addition of CpG-PEG-*b*-PLGA NPs to OVA-PS NP formulation induced modest CD8+ T-cell responses, comparable to that of the OVA-PS NP immunization. Soluble OVA alone also produced a modest response to the SIINFEKL epitope. The OVA-PEG-*b*-PLGA NPs did not induce IFN-γ T-cell responses to SIINFEKL, either alone or with the addition of CpG-PEG-*b*-PLGA NPs.

**Figure 5:**
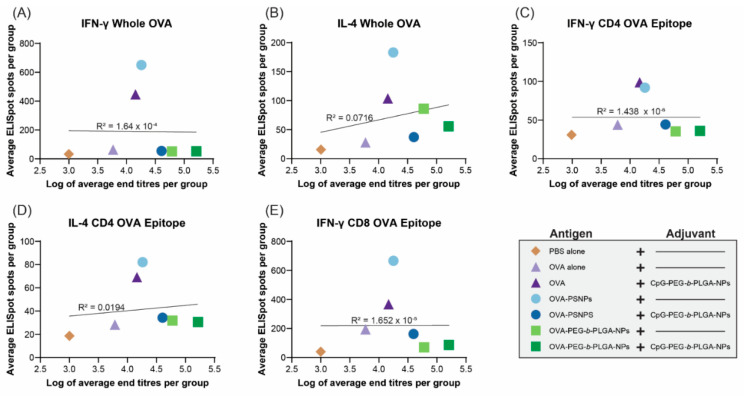
Correlations between cytokine levels and antibody end titers at day 28. C57BL/6 mice were immunized twice, two weeks apart, intradermally at the base of the tail with the following formulations; PBS alone (Naïve), OVA alone, OVA + CpG-PEG-*b*-PLGA NPs, OVA-PS NPs, OVA-PS NPs + CpG-PEG-*b*-PLGA NPs, OVA-PEG-*b*-PLGA NPs, or OVA-PEG-*b*-PLGA NPs + CpG-PEG-*b*-PLGA NPs. All animals received 50 µg OVA. CpG-PEG-*b*-PLGA NP groups received 5 µg CpG. Graphs show linear regression correlations between average antibody end titer (log) and the average number of IFN-γ and IL-4 producing cells from each group to (**A**,**B**) whole OVA, (**C**,**D**) CD4+ T-helper OVA epitope (ISQAVHAAHAEINEAGR), and (**E**) CD8+ cytotoxic T-cell OVA epitope (SIINFEKL).

Given the robust antibody responses and significant induction of IL-4 production from T-cells in the PEG-b-PLGA NP-immunized mice with some outlier high responders, we examined if there was a correlation between antibody titers and the level of cytokine producing cells from the ELISpot assay. Results from D28 samples were used for this analysis, as it was the first time point when the antibody levels rose and stayed relatively stable over time (up to Day 120 in some of the vaccinated groups). The same cohort of mice that were measured for T-cell cytokine production were also tested for antibody levels and their IgG titers measured at Day 28 were correlated. Figure 5 shows that there were no correlations observed between the number of IFN-γ or IL-4 producing T-cells in the spleen and serum antibody titers in the same animals at Day 28.

## 4. Discussion

Vaccine delivery systems offer an effective way to increase specific immune responses, including NP vaccine carriers. NPs as a vaccine delivery carrier offer several advantages to enhance immune responses by targeting antigen delivery, modulating antigen presentation, and tailoring the activation of immune cells. This study assesses the vaccine response to biodegradable PEG-*b*-PLGA NPs as a carrier for model antigen OVA and for CpG ODN 1018 adjuvant using an experimental benchmark NP formulation (40–50 nm PS NPs) as a control. This PS NP formulation has been shown to induce high levels of T-cells (both CD4+ T-cells and CD8+ T-cells) as well as antibody responses. Compared with a traditional adsorption-based adjuvant system, alum particles, OVA-conjugated PS NPs were superior in both CD8+ T cell responses and antibody titers [32]. Similar results were shown in our recent study with MSP4/5 (blood stage malaria antigen) conjugated NPs where PS NPs-MSP4/5 induced higher IFN-γ T-cell responses and comparable IL-4 T-cell responses compared to MSP4/5 immunized with alum [8]. There were also no differences in the total IgG or IgG1 responses when comparing PS NPs-MSP4/5 and alum adjuvanted MSP4/5 vaccines, but PS NPs-MSP4/5 induced higher IgG2a and IgG2b responses compared to alum. These studies indicate that antigen-conjugation to NPs inflict minimal or negligible modification of the protein antigen, and this strategy represents an effective approach to enhance antigen immunogenicity.

Both OVA-PS NPs and OVA-PEG-*b*-PLGA NPs induced significant IgG antibody responses and both required a boost immunization to reach high antibody titers. Promisingly, after the boost immunization substantial antibody levels remained stable over time (up to Day 120) (Figure 2). The OVA-PEG-*b*-PLGA NPs also produced a more consistent, albeit more moderate, antibody response in comparison to the OVA-PS NPs, which induced either markedly high responders or no responders (Figure 2D). A transient and low level of response was observed in animals immunized with OVA antigen alone. Such responses have been previously observed in studies where OVA has some degree of natural or artificially induced aggregation [33]. OVA-PS NPs induced high levels of IFN-γ producing CD4+ and CD8+ T-cells, as well as IL-4 producing T-cells. By contrast, OVA-PEG-*b*-PLGA NPs preferentially elicited IL-4 T-cell responses, with little to no induction of IFN-γ producing CD4+ or CD8+ T-cells. The preferential induction of IL-4 producing T-cells and antibody responses, in the absence of IFN-γ producing cells, points to the potential utility of these NPs for use in populations where the induction of Th1 cells or CD8+ T-cells promoting a consequent increased systemic propensity to this type of immunity may be deleterious, for example, in autoimmune patients [5,34], or individuals with cerebral malaria or other Th1 or CD8+ T-cell mediated pathologies.

Most NP-based vaccine carriers are not self-adjuvanting and require the incorporation of additional mechanisms or adjuvants to elicit high immune responses. On the basis of recent studies suggesting intracellular TLR9 signaling via NPs can selectively enhance different types of immunity [22], we constructed CpG ODN 1018 surface conjugated biodegradable NPs, CpG-PEG-*b*-PLGA NPs. These were 30 nm in size, with an expected 0.147 µg CpG/nm^2^ density content, which suggests they would preferentially adjuvant Th2 immunity [22]. Immunizing mice with OVA-PEG-*b*-PLGA NPs or OVA-PS NPs together with CpG-PEG-*b*-PLGA NPs as an adjuvant consistently increased the antibody responses. For both groups, the addition of CpG-PEG-*b*-PLGA NPs increased the antibody end titers following two immunizations on D49 and at endpoint on D120 for OVA-PEG-*b*-PLGA NPs, and from D28 through D120 for OVA-PS NPs. Moreover, CpG-PEG-*b*-PLGA NPs increased in antibody production for the OVA alone group, especially on D49 and D120, suggesting potential utility for this NP adjuvant both for protein antigen and for antigens formulated as NPs. Addition of CpG-PEG-*b*-PLGA NPs did not substantially alter either the magnitude or the type of T-cell immune responses generated by any vaccine formulation. This observation may be due to CpG-PEG-*b*-PEG NPs being a minor constituent of the NP vaccine mixture, limiting the likelihood of successful colocalization of separate antigen and TLR adjuvant NPs in the same endosome/lysosomal compartment in target APCs necessary for preferred processing, APC activation, and antigenic presentation [35]. Neither IFN-γ nor IL-4 production by CD8+ or CD4+ T-cells correlated with antibody titers, indicating that these responses were independently elicited.

The responses elicited by PEG-*b*-PLGA NPs and PS NPs can be further explained by differences in degradability, antigen/adjuvant persistence, and nanoparticle colloidal stability. These chemical and physical differences would result in downstream differences in lymph node targeting, APC uptake, antigen-processing and presentation [36]. Hydrophobicity is thought to be a potent danger signal since exposed hydrophobic residues act as universal damage-associated molecular patterns (DAMPs) that normally are only present in necrotic cell death and pathogens [37]. Hydrophobicity leads to changes in the composition of the protein corona at the injection site and while draining to the lymph node [38]. The hydrophobic surface of PS NPs and their associated protein corona lead to enhanced interaction with immune cells while potentially diminishing lymph node targeting abilities [27,30,36,39,40]. PEGylated PEG-*b*-PLGA NPs have enhanced lymphatic drainage and slight diminishment in immune cell uptake [10]. OVA conjugated to the PS NP surface will likely denature due to the hydrophobic surface while OVA conjugated to PEG-*b*-PLGA NPs will retain their tertiary structure after conjugation, leading to differences in downstream antigen processing. Furthermore, the hydrophobic PS NPs have poor colloidal stability without addition of surfactants compared to PEG-*b*-PLGA NPs that are highly stable in solution form leading to differences in biodistribution after administration [41]. The degradability plays a large role in elicited response, with the PS NPs having longer persistence and presentation of antigen/adjuvant cues compared to biodegradable PEG-*b*-PLGA NPs. Lastly, subtle differences in the nanoparticle surface and rugosity have been shown to play a role in in controlling how nanoparticles interact with different APCs [42]. All these differences in physicochemical properties between PS NPs and PEG-*b*-PLGA NPs contribute to differing Th1/Th2 skewing activity and warrant additional in-depth investigation to find causal structure–function relationships.

Together these data show potential utility for CpG-PEG-*b*-PLGA NPs as an adjuvant to elicit more consistent and sustained antibody responses. Additionally, it suggests further exploration may be warranted for PEG-*b*-PLGA NPs as an antigen carrier for antibody production and the elicitation of IL-4 producing T-cells in the absence of IFN-γ or CD8+ T-cell responses. More studies will be required to determine if this Th2 skewed response is implicit to the PEG-*b*-PLGA platform or if Th1 immunity can be likewise induced by adding CpG and OVA to the same NP surface or increasing the densities of the adjuvant/antigen cues as demonstrated in other NP systems [22].

## Supplementary Materials

The following are available online at https://www.mdpi.com/2076-393X/8/2/261/s1, Figure S1: Fabrication and characterization of PEG-*b*-PLGA NPs; Figure S2: Nanovaccine antibody responses over 120 days; and Table S1: Statistical analysis of end titers over 120 days.
