# Supplementary material for: Biodegradable PLGA-b-PEG Nanoparticles Induce T Helper 2 (Th2) Immune Responses and Sustained Antibody Titers via TLR9 Stimulation
Source: Vaccines (Basel). 2020 May 29;8(2):261. doi: 10.3390/vaccines8020261 (PMC7349924; doi:10.3390/vaccines8020261)
Supplement: Supplementary file 1 [file vaccines-08-00261-s001.pdf]

vaccines

Article

Biodegradable PLGA-b-PEG nanoparticles induce T helper 2 (Th2) immune responses and sustained antibody titers via TLR9 stimulation

Kirsty L. Wilson[1,2,†], Gregory P. Howard[3,4,†], Heather Coatsworth[5], Rhoel R. Dinglasan[5,*], Hai-Quan Mao[3,4,6,*], and Magdalena Plebanski[1,2,*]

[1] School of Health and Biomedical Sciences, Royal Melbourne Institute of Technology (RMIT) University, Melbourne, Victoria 3084, Australia; kirsty.wilson2@rmit.edu.au
[2] Department of Immunology and Pathology, Monash University, Melbourne, Victoria 3181, Australia
[3] Department of Biomedical Engineering, Johns Hopkins School of Medicine, Baltimore 21205, USA
[4] Institute for NanoBioTechnology, Johns Hopkins University, Baltimore 21218, USA
[5] Emerging Pathogens Institute, Department of Infectious Diseases & Immunology, College of Veterinary Medicine, University of Florida, Gainesville, FL 32611, USA
[6] Department of Materials Science and Engineering, Whiting School of Engineering, Johns Hopkins University, Baltimore 21218, USA
† Authors contributed equally to this work.
* Correspondence: R.R.D., rdinglasan@epi.ufl.edu; H.Q.M., hmao@jhu.edu; M.P., magdalena.plebanski@rmit.edu.au .

Received: date; Accepted: date; Published: date

Abstract: Sustained immune responses, particularly antibody responses, are key for protection against many endemic infectious diseases. Antibody responses are often accompanied by T helper (Th) cell immunity. Herein we study small biodegradable poly(ethylene glycol)-*b*-poly(lactic-co-glycolic acid) nanoparticles (PEG-*b*-PLGA NPs, 25–50 nm) as antigen- or adjuvant-carriers. The antigen carrier function of PEG-*b*-PLGA NPs was compared against an experimental benchmark polystyrene nanoparticles (PS NPs, 40–50 nm), both conjugated with the model antigen ovalbumin (OVA-PS NPs, and OVA-PEG-*b*-PLGA NPs). The OVA-PEG-*b*-PLGA NPs induced sustained antibody responses to Day 120 after two immunizations. The OVA-PEG-*b*-PLGA NPs as a self-adjuvanting vaccine further induced IL-4 producing T-helper cells (Th2), but not IFN-γ producing T-cells (Th1). The PEG-*b*-PLGA NPs as a carrier for CpG adjuvant (CpG-PEG-*b*-PLGA NPs) were also tested as mix-in vaccine adjuvants comparatively for protein antigens, or for protein-conjugated to PS NPs or to PEG-*b*-PLGA NPs. While the addition of this adjuvant NP did not further increase T-cell responses, it improved the consistency of antibody responses across all immunization groups. Together these data support further development of PEG-*b*-PLGA NPs as a vaccine carrier, particularly where it is desired to induce Th2 immunity and achieve sustained antibody titers in the absence of affecting Th1 immunity.

Keywords: Nanoparticle, adjuvant, vaccine, antibody, immune response

---

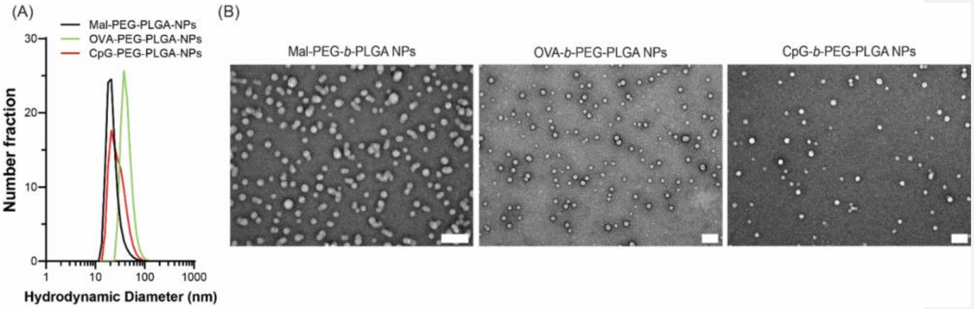

Figure S1. Fabrication and characterization of PEG-*b*-PLGA ~~PLGA-*b*-PEG~~ NPs. (A) DLS measurements with number average hydrodynamic diameters for unconjugated Maleimide-PEG-*b*-PLGA NPs and surface conjugated OVA-PEG-*b*-PLGA NPs and CpG-PEG-*b*-PLGA NPs. DLS measurements confirmed that the conjugation was successful. (B) Transmission electron microscopy confirmed the average hydrodynamic diameters and size distributions for the Mal-PEG-*b*-PLGA NPs, OVA-PEG-*b*-PLGA NPs, and CpG-PEG-*b*-PLGA NPs to be 28-, 44-, and 29-nm, respectively. Scale bar = 100 nm.

Formatted: Font: Not Bold

Formatted: Font: Italic

Formatted: Font: Not Bold

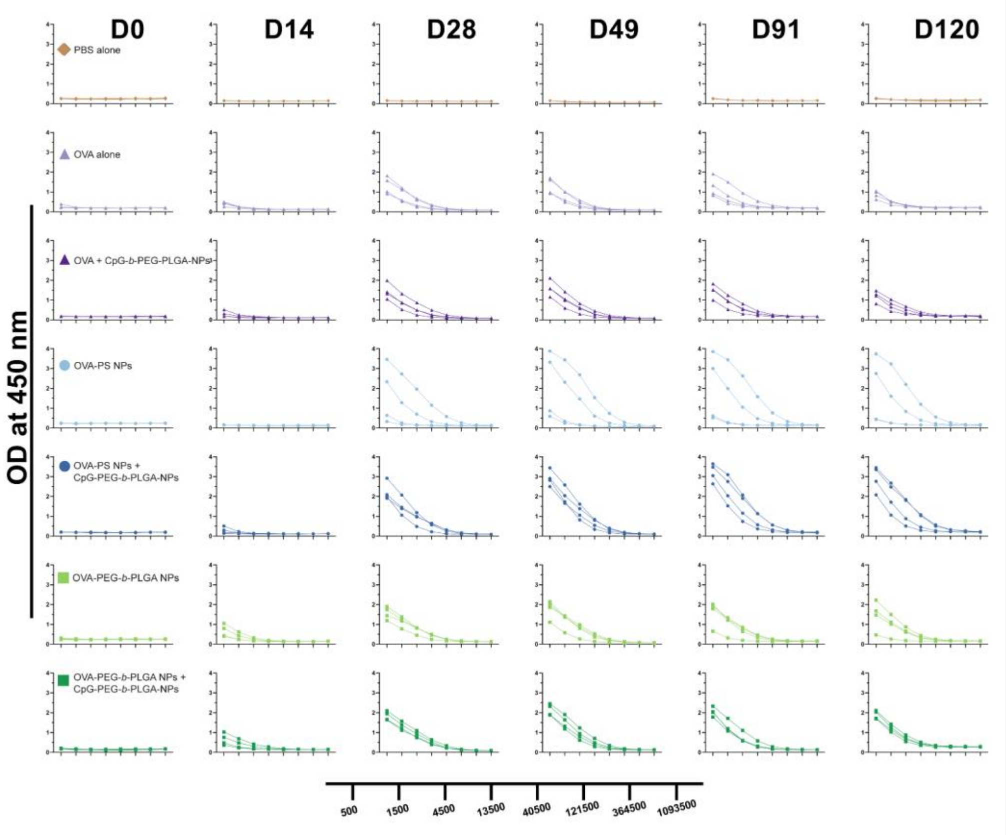

Figure S2. Nanovaccine antibody responses over 120 days. C57BL/6 mice were immunized twice, two weeks apart, intradermally at the base of the tail with the following formulations; PBS alone (Naïve), OVA alone, OVA + CpG-PEG-*b*-PLGA NPs, OVA-PS NPs, OVA-PS NPs + CpG-PEG-*b*-PLGA NPs, OVA-PEG-*b*-PLGA NPs, or OVA-PEG-*b*-PLGA NPs + CpG-PEG-*b*-PLGA NPs. All animals received 50 µg OVA. CpG-PEG-*b*-PLGA NP groups received 5 µg CpG. Graphs show individual mice responses for each group (rows) at each time point (columns).

Formatted: Font: Not Bold

	D0	D14	D28	D49	D91	D120
Naive	*n.s.*	a	a	a	a	a
OVA	*n.s.*	b	b	b	b	b
OVA+CpG-PLGA-PEG-NPs	*n.s.*	b	b	bc	b	bc
OVA-PSNPs	*n.s.*	ac	b	bc	b	bc
OVA-PSNPS+CpG-PLGA-PEG-NPs	*n.s.*	bc	b	c	b	c
OVA-PLGA-PEG-NPs	*n.s.*	a	b	bc	b	bc
OVA-PLGA-PEG-NPs+CpG-PLGA-PEG-NPs	*n.s.*	c	b	bc	b	c

Formatted Table

	D14	D28	D49	D91	D120
Naive	a	a	a	a	a
OVA	b	b	b	b	b
OVA + CpG-PEG-*b*-PLGA NPs	b	b	bc	b	bc
OVA-PS NPs	ac	b	bc	b	bc
OVA-PS NPs + CpG-PEG-*b*-PLGA NPs	bc	b	c	b	c
OVA-PEG-*b*-PLGA NPs	a	b	bc	b	bc
OVA-PEG-*b*-PLGA NPs + CpG-PEG-*b*-PLGA NPs	c	b	bc	b	c

Table S1. Statistical analysis of end titers over 120 days. End point titer data from Figure 2 were analyzed and treatments without a common letter were found to be statistically significant ($\alpha = 0.05$) using a Kruskal-Wallis test with Mann-Whitney pairwise post-hoc comparisons. No significant differences were detected between any treatment group at day 0; however, significant differences were noted between treatment groups at day 14, 49 and 120.

Commented [GH1]: This figure is a table. Renamed Figure S3 to Table S1 here and in manuscript.
